# Surgical approach to a left ventricular myxoma guided by transesophageal and epicardial dual-view echocardiography: a case report

**DOI:** 10.1186/s40792-016-0224-8

**Published:** 2016-09-08

**Authors:** Azumi Hamasaki, Kazunori Ishikawa

**Affiliations:** 1 Department of Cardiovascular Surgery, Cardiovascular Center, Sendai Kosei Hospital, 4-15, Hirose-machi, Aoba-ku, Sendai, 980-0873 Japan; 2 Second Department of Surgery, Yamagata University Faculty of Medicine, 2-2-2, Iida-Nishi, Yamagata, 990-9585 Japan; 3 Division of Cardiovascular Surgery, Maebashi Red Cross Hospital, 3-21-36, Asahi-cho, Maebashi, 371-0014 Japan

**Keywords:** Left ventricular myxoma, Intraoperative echocardiography, Surgical approach

## Abstract

**Background:**

Left ventricular myxoma is a rare benign cardiac neoplasm. Surgical excision is the treatment of choice, and complete removal is mandatory to prevent late recurrence.

**Case presentation:**

Here, we report a case of myxoma originating from the anterolateral wall of the left ventricle with a very short stalk. Accordingly, the transaortic and transmitral approaches were considered inadequate; therefore, a transventricular approach was adopted. To minimize the incidence of complications associated with a left ventriculotomy, its length was designed to be as short as possible. To plan a proper ventriculotomy, measurements were taken by the combined use of transesophageal echocardiography and epicardial direct echocardiography.

**Conclusions:**

This method provided a good guide without being a complicated technique. The tumor and its attachment were clearly visualized and completely resected in an en bloc fashion. No recurrence has occurred to date.

## Background

Myxomas, the most common cardiac neoplasms, are usually detected in the left atrium. Left ventricular myxomas account for only a small percentage of cardiac myxomas [1]. To prevent recurrence, full excision is important. To avoid left ventriculotomy, many of these tumors are resected via a transmitral or transaortic approach [2, 3]. However, because of anatomical limitations, a left ventriculotomy is required in some cases to ensure complete resection. An intraoperative evaluation by the combined use of transesophageal and epicardial direct echocardiography is useful for planning the incision site of a left ventriculotomy.

## Case presentation

A 66-year-old man was referred to our hospital with the diagnosis of a left ventricular mass detected incidentally during transthoracic echocardiography that was performed prior to a cholecystectomy. He had no cardiac symptoms or history of embolism. On physical examination, he had normal heart sounds without any cardiac murmurs. The results on chest radiography, electrocardiography, and blood examinations were normal. Transthoracic echocardiography revealed a mobile mass, 16 mm in diameter, originating from the anterolateral wall of the left ventricle. Its stalk was short, and the tumor was directly attached to the ventricular wall. The diameter of the left ventricle was 52 mm in the diastolic phase and 30 mm in the systolic phase. The ejection fraction (EF) of the left ventricle was estimated as 80 %. Computed tomography showed a homogeneous 20-mm-diameter tumor that originated from the anterolateral wall of the left ventricle with an extremely short stalk.

**Fig. 1:**
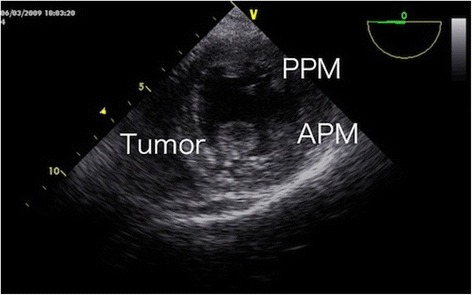
Intraoperative transesophageal echocardiography showing a round tumor originating from the anterolateral wall of the left ventricle. *APM*, anterior papillary muscle; *PPM*, posterior papillary muscle

**Fig. 2:**
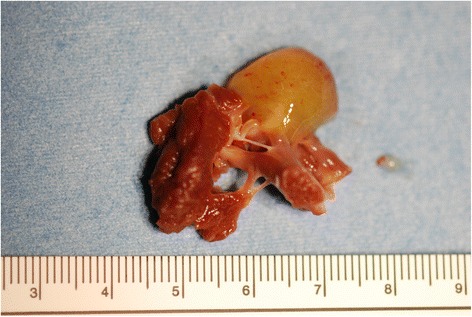
Macroscopic findings of the resected specimen. The tumor originated at the trabecular muscle. A good surgical margin (>5 mm) was achieved

Under general anesthesia, the heart was exposed through a median thoracotomy. The pericardium was retracted by Lima suture [4]. The tumor was observed in detail via transesophageal echocardiography (TEE). Tumor location, its relation to the coronary arteries in particular, was estimated by gentle pressure placed on the left ventricular wall by the surgeon’s finger. The origin of the tumor was detected to be on the free wall of the left ventricle between the first diagonal and obtuse marginal branches (Fig. 1). The incision line was carefully planned under echo-guidance and then cardiopulmonary bypass through bicaval and aortic cannulation was instituted. The heart was arrested with antegrade tepid blood cardioplegia. The final location of the incision was determined by epicardial echocardiography focused on the surgical margin. A 3-cm-long ventriculotomy was placed between the first diagonal and obtuse marginal branches along the planned incision line. A gelatinous 18-mm mass was directly visualized and found to originate from the trabecular muscle. The distance from the incision line to the edge of the stalk was 5 mm. The tumor was completely resected with a >5-mm margin of attached trabecular muscle (Fig. 2).

On a subsequent histopathological examination, typical features of myxoma were demonstrated, a good surgical margin >5 mm was achieved, and there was no evidence of invasion in the cross-section of the resected myocardium. The ventriculotomy was closed by four mattress sutures and a running 4-0 polyvinylidenefluoride suture (Asflex; CrownJun Kono Corp., Tokyo) with underlying Teflon felt strips (CrownJun Kono Corp., Tokyo). The postoperative course was uneventful, and no evidence of recurrence or arrhythmia has been seen in the 24 months since the operation. Postoperative cardiac function was estimated by echocardiography prior to discharge from the hospital and at 6 months after the operation. The diastolic and systolic diameters of the left ventricle were 50 and 26 mm, respectively, at discharge, and 51 and 30 mm, respectively, at 6 months after the surgery. The EF of the left ventricle at discharge and 6 months after the surgery was 85 and 75 %, respectively.

### Discussion

Primary cardiac tumors are rare. Nearly 75 % are benign, and most of these benign heart tumors are myxomas. Approximately 75 % of cardiac myxomas originate from the left atrium; only 3–4 % are found in the left ventricle [1]. Myxomas are histopathologically benign; however, they tend to recur. The major surgical concern is preventing recurrence. Complete extirpation with precise removal of the base under a clear surgical view is required. In the case of myxomas originating from the left ventricle, the surgical approach is important. A transaortic or transmitral approach is generally employed for their removal [2, 3]. A transaortic approach would be useful for myxomas located in the left ventricular outflow tract versus the transmitral approach for those in posterior locations. Many of the myxomas originating from the left ventricle can be removed by those approaches. However, in cases of myxomas with a short stalk, or myxomas located at the apical region or subvalvular region, surgical manipulation by the approaches mentioned above should be performed under an inadequate surgical view.

Greco et al. reported an approach combined with video-assisted cardioscopy for myxoma at the apex region [5]. For subvalvular myxomas, Talwalker et al. reported an approach using mobilization of the anterior leaflet of the mitral valve [6]. These alternatives may have advantages in certain limited cases, but are too complicated to be standard approaches. Even though a transventricular approach has potential complications, such as dysfunction of the left ventricle or postoperative ventricular arrhythmia, this approach provides a good surgical view that enables excision of both the tumor and its stalk in an en bloc fashion.

In our patient, the approach route was planned preoperatively. Because the tumor was located on the anterolateral wall of the left ventricle and had a short and wide stalk and the diameter of the aortic annulus (estimated as 21 mm) was relatively small, we concluded that the transaortic and transmitral approaches were inadequate. We estimated that the transaortic view via a relatively small aortic annulus might be insufficient to achieve complete resection. We also examined the transmitral approaches including superior septal and Dubost incision. We concluded that a competent surgical field except beyond the tumor might be provided by these approaches. The incidence of incomplete surgical margin at the apical edge might be considered. Since our highest priority should be to achieve complete tumor resection, we decided to select a transventricular approach. To minimize the incidence of complications associated with a left ventriculotomy, the length of the ventriculotomy was designed to be as short as possible. A proper ventriculotomy was planned according to measurement by the combined use of TEE [7] from the inside and epicardial direct echocardiography from the outside. This method provided a good guide without being complicated. The tumor and its attachment were exposed under good visualization and completely resected in an en bloc fashion. No recurrence has occurred to date.

## Conclusions

Here, we described a rare case of cardiac myxoma originating from the left ventricular free wall and presented our simple technique for planning the surgical approach using intraoperative echo guidance.

## Consent

Written informed consent was obtained from the patient for publication of this case report and any accompanying images.

## Abbreviations

EF: Ejection fraction
TEE: Transesophageal echocardiography
